# Supplementary material for: A subgroup of light-driven sodium pumps with an additional Schiff base counterion
Source: Nat Commun. 2024 Apr 10;15:3119. doi: 10.1038/s41467-024-47469-0 (PMC11006869; doi:10.1038/s41467-024-47469-0)
Supplement: Supplementary file 1 — Supplementary Information [file 41467_2024_47469_MOESM1_ESM.pdf]

SUPPLEMENTARY INFORMATION

Supplementary Figures

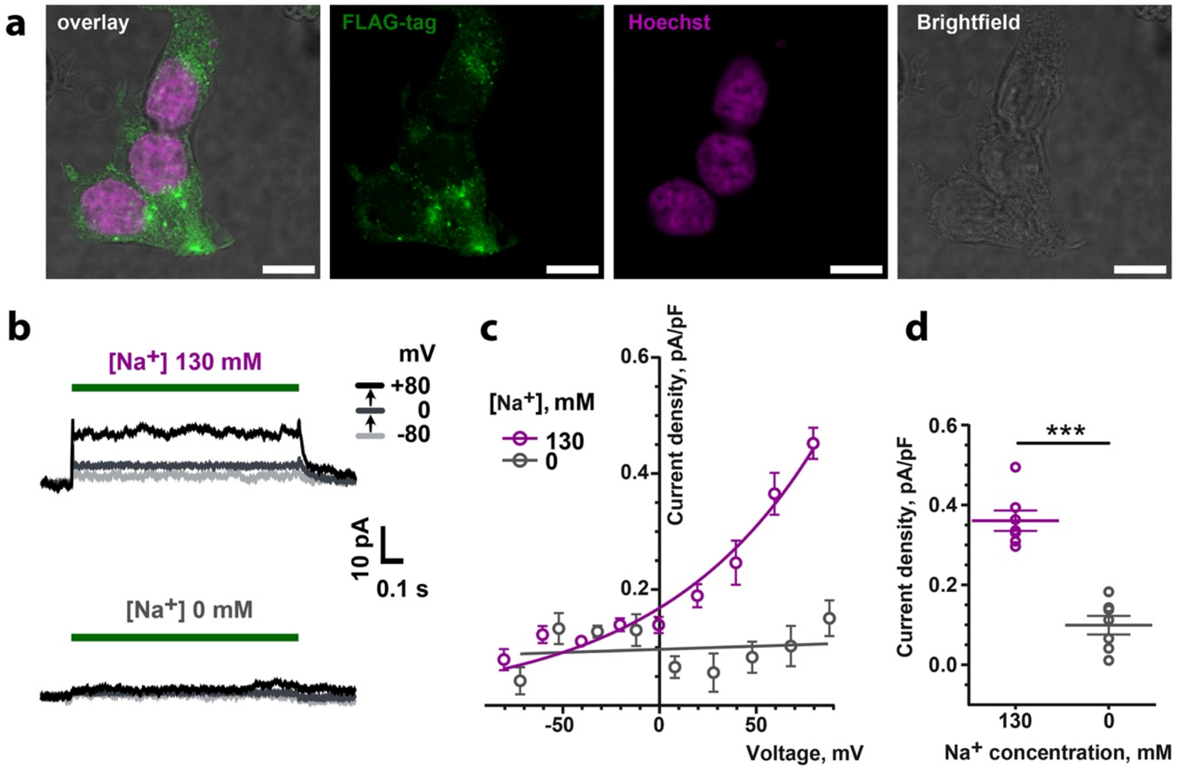

Fig. S1. Functional characterization of C2C1-*Er*NaR-TS-FLAG in HEK293T cells. a. Representative confocal images of HEK293T cells expressing C2C1-*Er*NaR-TS-FLAG. The AlexaFluor488-conjugated FLAG-tag antibody is shown in green; nucleus stain Hoechst 33342 - in magenta. Scale bars, 10 µm. The localization was similar in at least 6 representative confocal images from 2 independent transfections. **b.** Representative photocurrents of C2C1-*Er*NaR-TS-FLAG recorded from HEK293T cells at 130 mM (top) and 0 mM (bottom) intracellular $[Na^+]_i$ and pH_i 7.5. **c.** Voltage dependence of the stationary photocurrents of C2C1-*Er*NaR-TS-FLAG at intracellular $[Na^+]_i$ 130 mM (magenta) and 0 mM (gray) and pH_i 7.5 (LJP-corrected; normalized to respective cell capacitance; mean ± SEM of n = 7 cells in each condition). **d.** Stationary photocurrents of C2C1-*Er*NaR-TS-FLAG at +60 mV, normalized to respective cell capacitance (mean ± SEM and individual data points). Data were extracted from the recordings at different intracellular $[Na^+]_i$ described in **c.** Normalized currents were analyzed using Mann-Whitney test (****P*=0.0006). All patch-clamp experiments were conducted at 110 mM extracellular $[Na^+]_e$, pH_e 7.5; LED light with maximum at 550 nm was applied for 1 s at 34.3 mW/mm² irradiance.

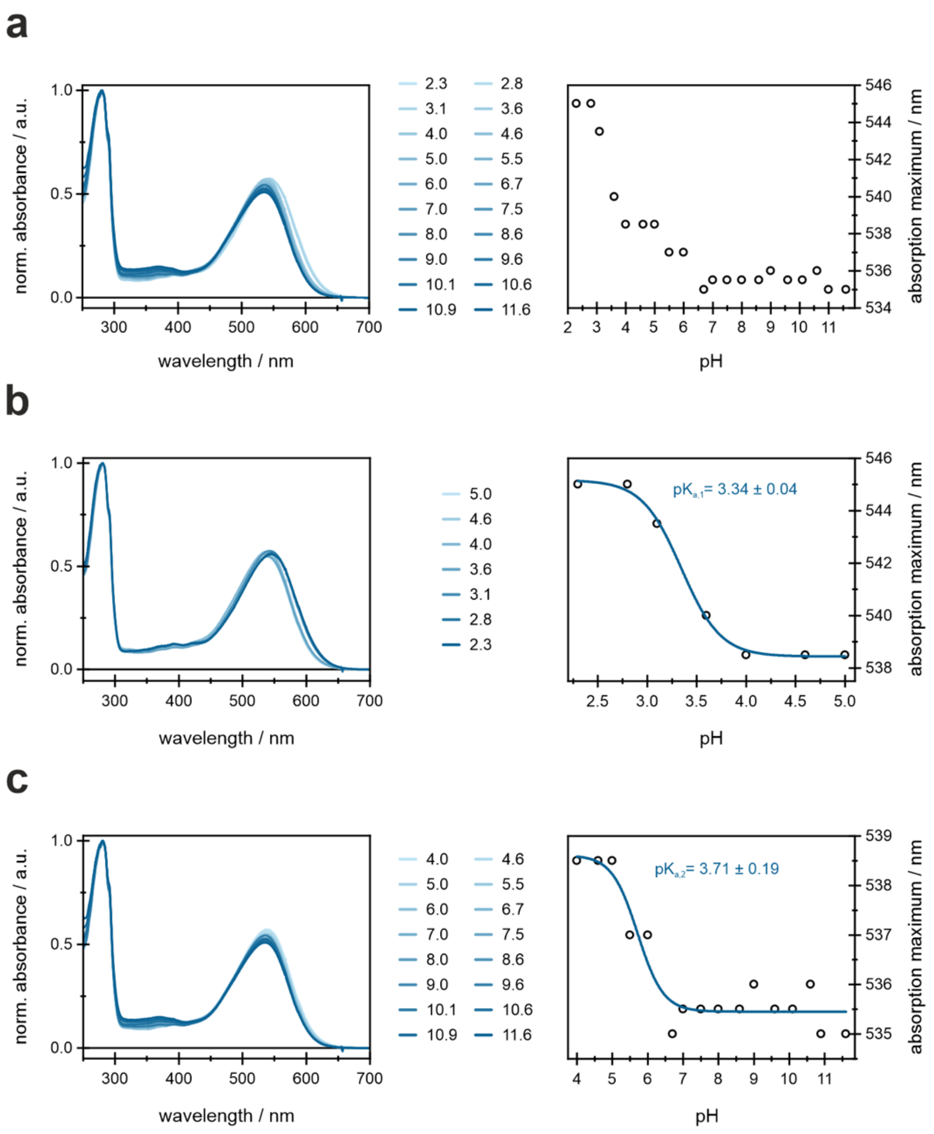

Fig. S2. pH titration of detergent-solubilized *Er*NaR. Normalized absorption spectra of *Er*NaR for **a.** the complete investigated pH range (2.3 – 11.6), **b.** the pH range that corresponds to pK$_{a,1}$(pH 2.3 – pH 5.0) and **c.** the pH range that corresponds to pK$_{a,2}$ (pH 5.0 – 11.6). The spectra have been normalized to the absorbance at 280 nm. Lowering the pH value is accompanied by a spectral blue-shift of the main absorption band. In **b** and **c**, the observed trends were fitted with the Boltzmann function to obtain the pK$_a$ values.

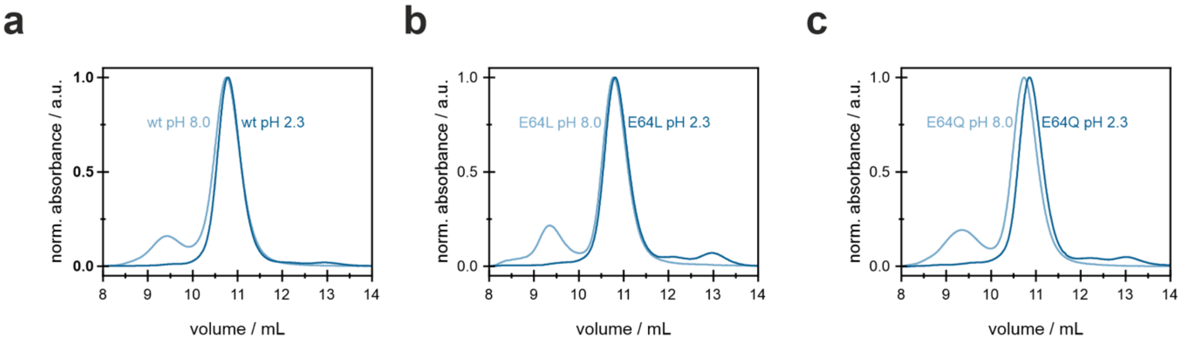

Fig. S3. Size-exclusion chromatography profiles of *Er*NaR and its mutants. a. Size-exclusion chromatography (SEC) profiles of the wild-type (WT) *Er*NaR at pH 2.3 (dark blue) and 8.0 (light blue). The absorbance was measured at 545 and 535 nm for pH 2.3 and 8.0, respectively. **b.** SEC profiles of the E64L variant of *Er*NaR at pH 2.3 (dark blue) and 8.0 (light blue). The absorbance was measured at 570 and 550 nm for pH 2.3 and 8.0, respectively. **c.** SEC profiles of the E64Q variant of *Er*NaR at pH 2.3 (dark blue) and 8.0 (light blue). The absorbance was measured at 546 and 525 nm for pH 2.3 and 8.0, respectively.

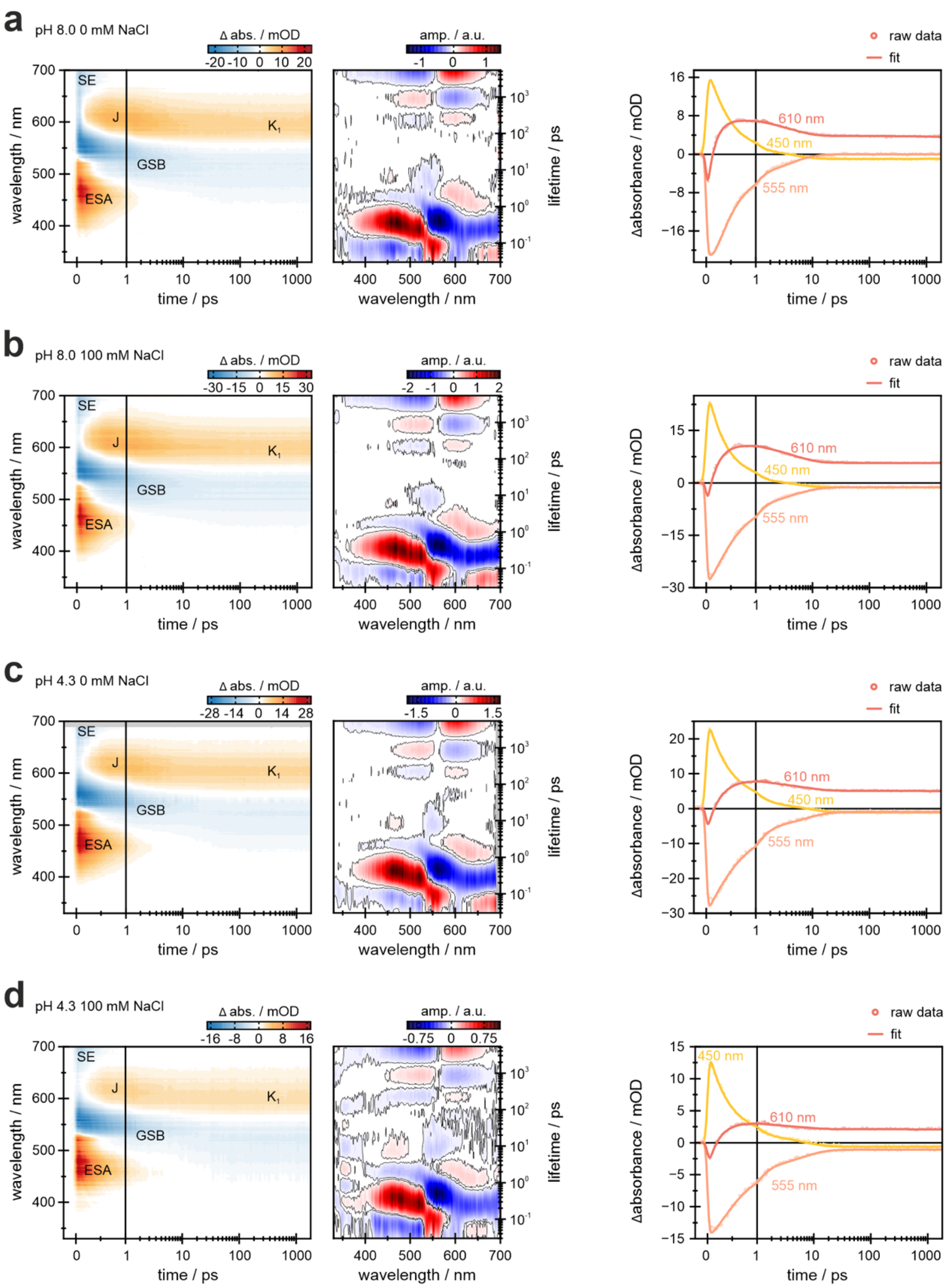

Fig. S4. Ultrafast transient absorption data of *Er*NaR at different conditions. 2D-contour plots (left) and lifetime distribution maps (LDM) of ultrafast transient absorption measurements of *Er*NaR at **a.** pH 8.0 0 mM NaCl, **b.** pH 8.0 100 mM NaCl, **c.** pH 4.3 0 mM NaCl and **d.** pH 4.3 100 mM NaCl. Additionally, transients representative for the different signals at 450, 555 and 610 nm are shown as raw data (dots) and the obtained fit (lines) of the respective transients (right).

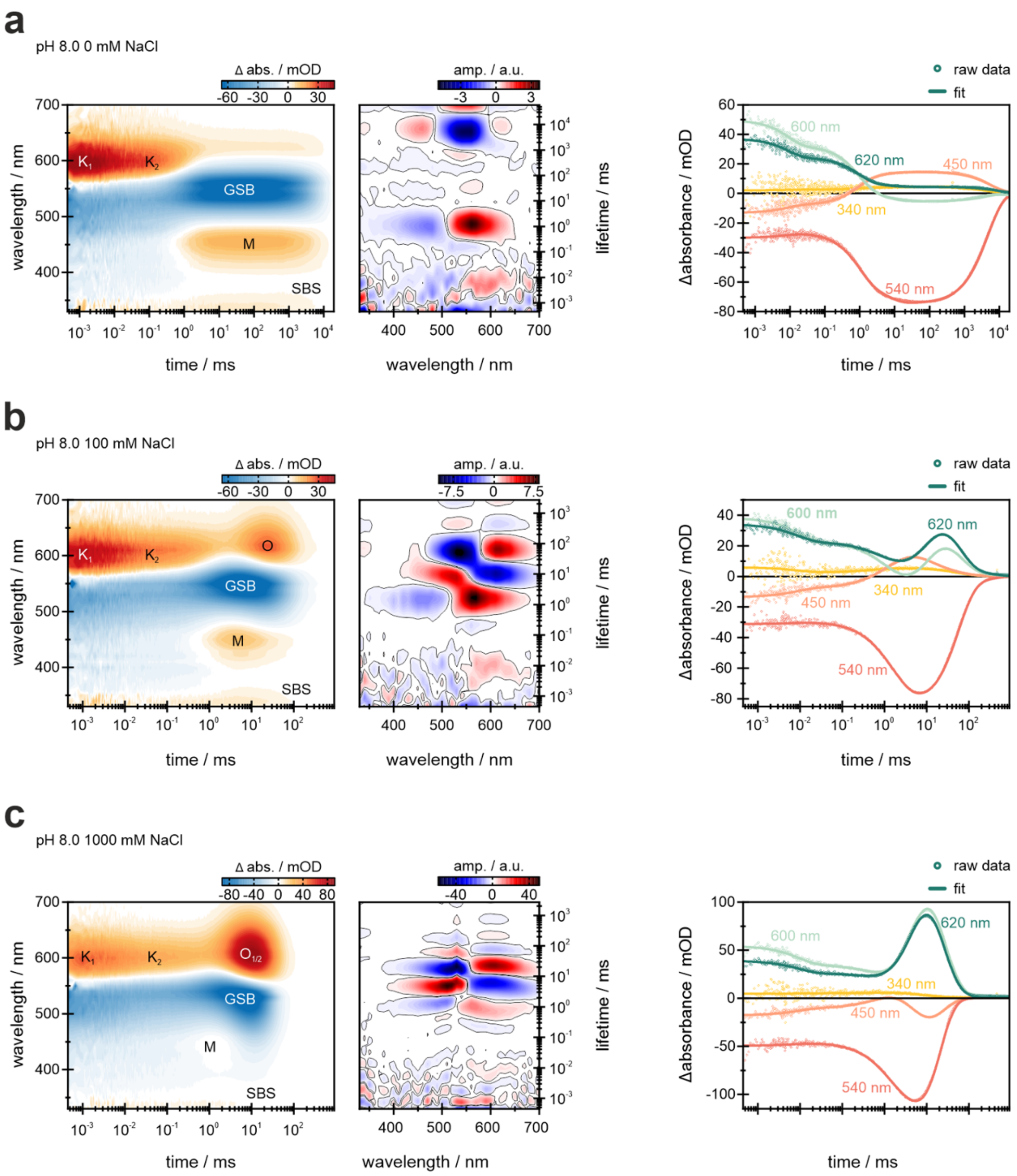

Fig. S5. Transient flash photolysis data of *Er*NaR at pH 8.0 with different NaCl concentrations. 2D-contour plots (left) and lifetime distribution maps (LDM) of transient absorption measurements of *Er*NaR with **a.** 0 mM NaCl, **b.** 100 mM NaCl and **c.** 1000 mM NaCl. Additionally, transients representative for the photocycle intermediates at 340 nm, 450 nm, 540 nm, 600 nm and 620 nm are shown as raw data (dots) and the obtained fit (lines) of the respective transients (right). Due to spectral similarity, the region of the O_1 and O_2 intermediates is indicated as $O_{1/2}$.

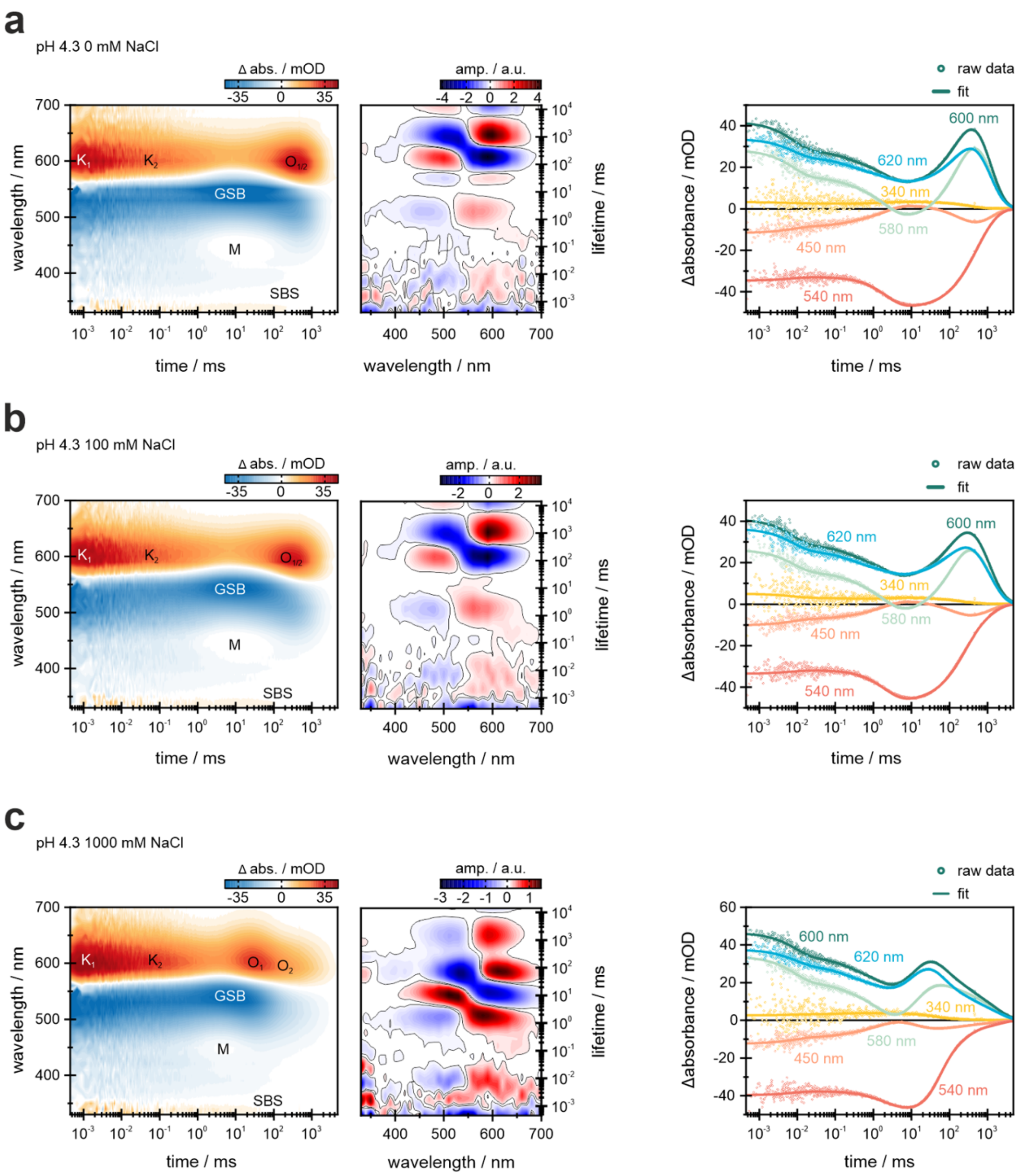

Fig. S6. Transient flash photolysis data of *Er*NaR at pH 4.3 with different NaCl concentrations. 2D-contour plots (left) and lifetime distribution maps (LDM) of transient absorption measurements of *Er*NaR with **a.** 0 mM NaCl, **b.** 100 mM NaCl and **c.** 1000 mM NaCl. Due to spectral similarity, the region of the O_1 and O_2 intermediates is indicated as $O_{1/2}$ in panels **a** and **b**. Additionally, transients representative for the photocycle intermediates at 340 nm, 450 nm, 540 nm, 580 nm, 600 nm and 620 nm are shown as raw data (dots) and the obtained fit (lines) of the respective transients (right).

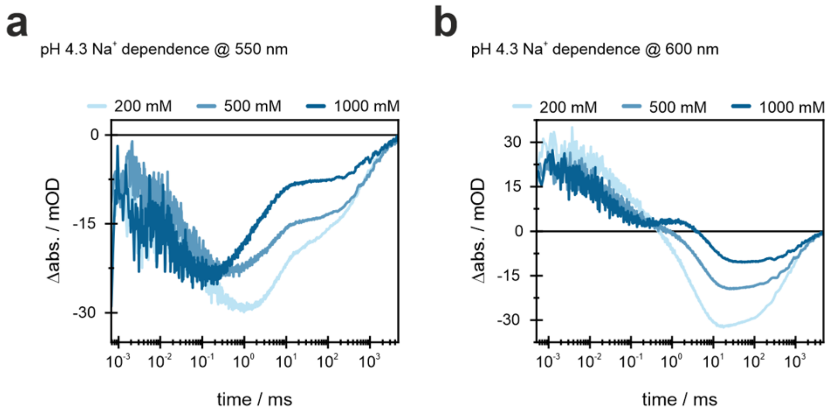

Fig. S7. Sodium concentration dependent transient flash photolysis data of KR2 at pH 4.3. Sodium concentration dependent measurements of the KR2 photocycle performed at probing wavelengths 550 nm (**a**) and 600 nm (**b**). Sodium concentrations 200 mM, 500 mM and 1000 mM NaCl were investigated.

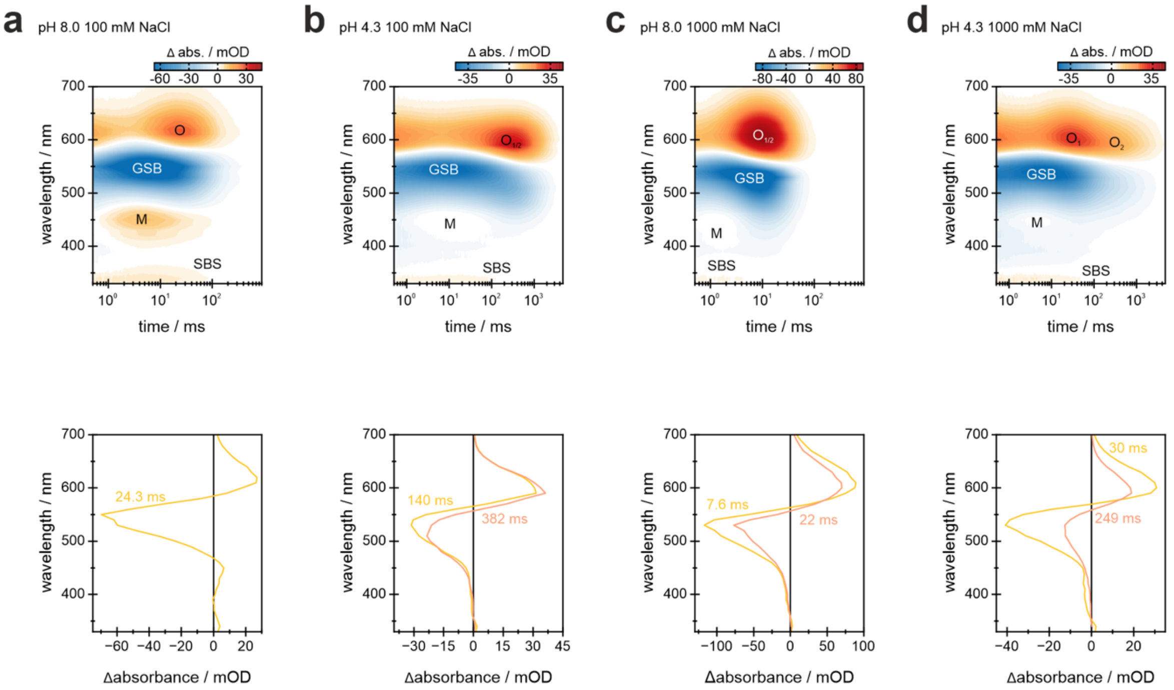

Fig. S8. Sodium and pH dependence of the spectral position of the O_1 and O_2 states. 2D-contour plots as well as transient spectra at specific time points describe both O intermediates of the flash photolysis measurements of *Er*NaR at **a** pH 8.0 100 mM NaCl, **b** pH 4.3 100 mM NaCl, **c** pH 8.0 1000 mM NaCl and **d** pH 4.3 1000 NaCl. The 2D-contour plots are shown from 0.5 ms onwards till the end of the measurement to focus on the O_1 and O_2 states. Due to spectral similarity, the region of the O_1 and O_2 intermediates is indicated as $O_{1/2}$ in panels **b** and **c**.

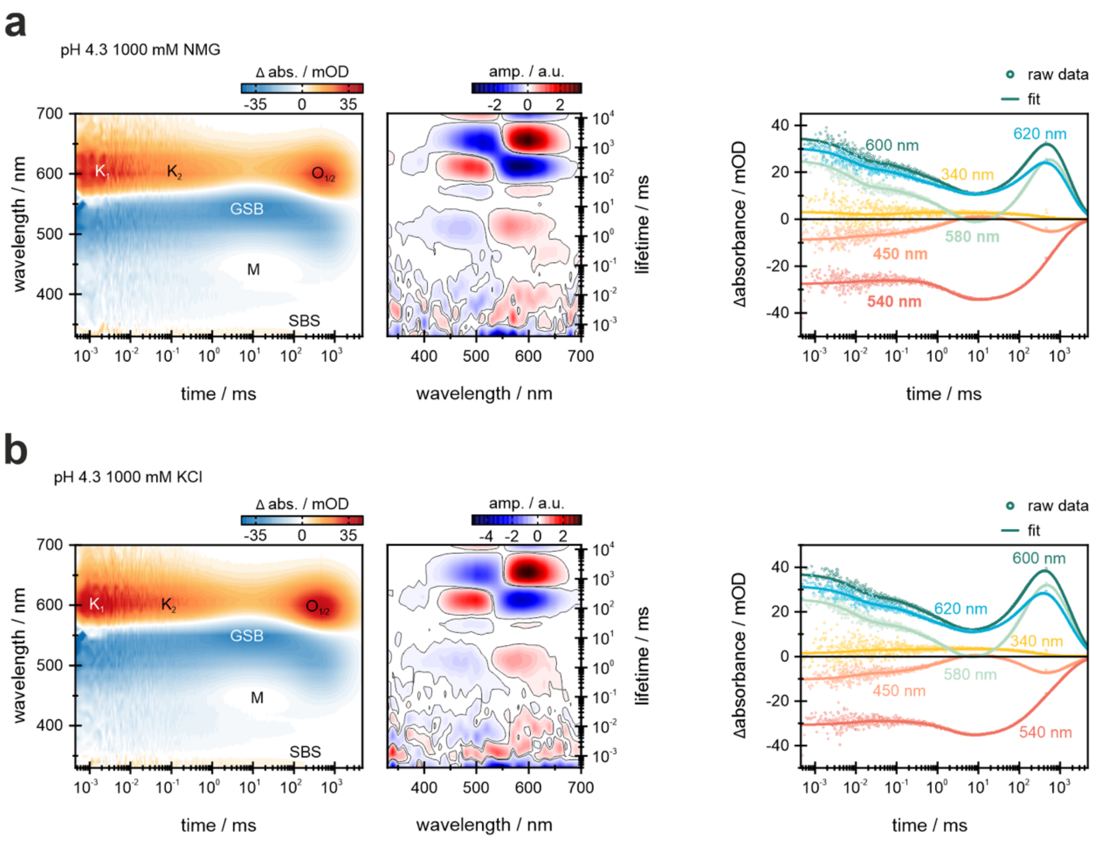

Fig. S9. Transient flash photolysis data of *Er*NaR at pH 4.3 in the presence of different ions. 2D-contour plots (left) and lifetime distribution maps (LDM) of transient absorption measurements of *Er*NaR with **a.** 1000 mM N-Methyl-D-glucamine (NMG) and **b.** 1000 mM KCl. Additionally, transients representative for the photocycle intermediates at **b** 340 nm, 450 nm, 540 nm, 600 nm and 620 nm and **b** 340 nm, 450 nm, 540 nm, 580 nm, 600 nm and 620 nm are shown as raw data (dots) and the obtained fit (lines) of the respective transients (right). Due to spectral similarity, the region of the O_1 and O_2 intermediates is indicated as $O_{1/2}$.

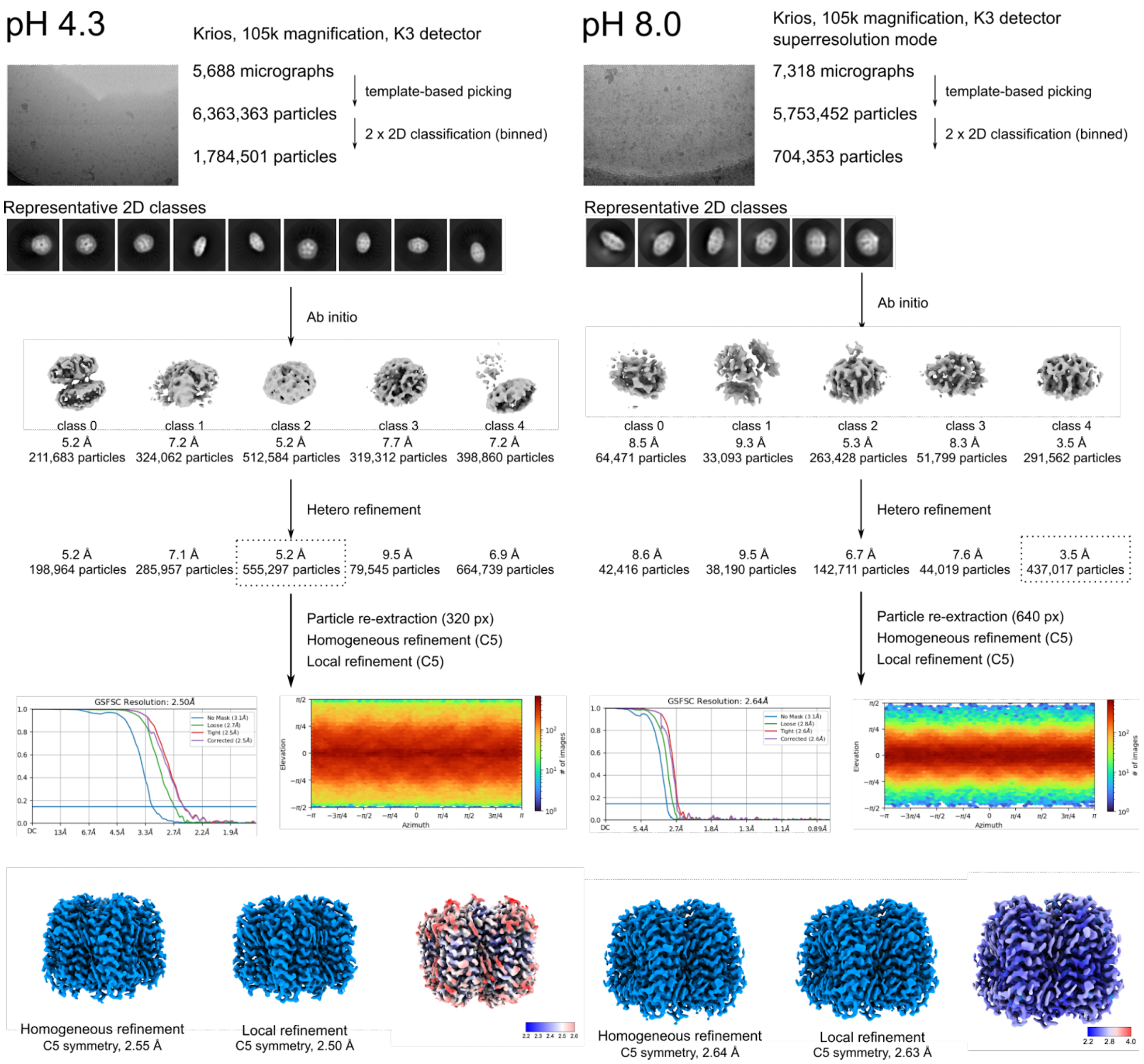

Fig. S10. Workflow for solving the *Er*NaR structure using cryoSPARC. Overall resolution and local resolution are shown.

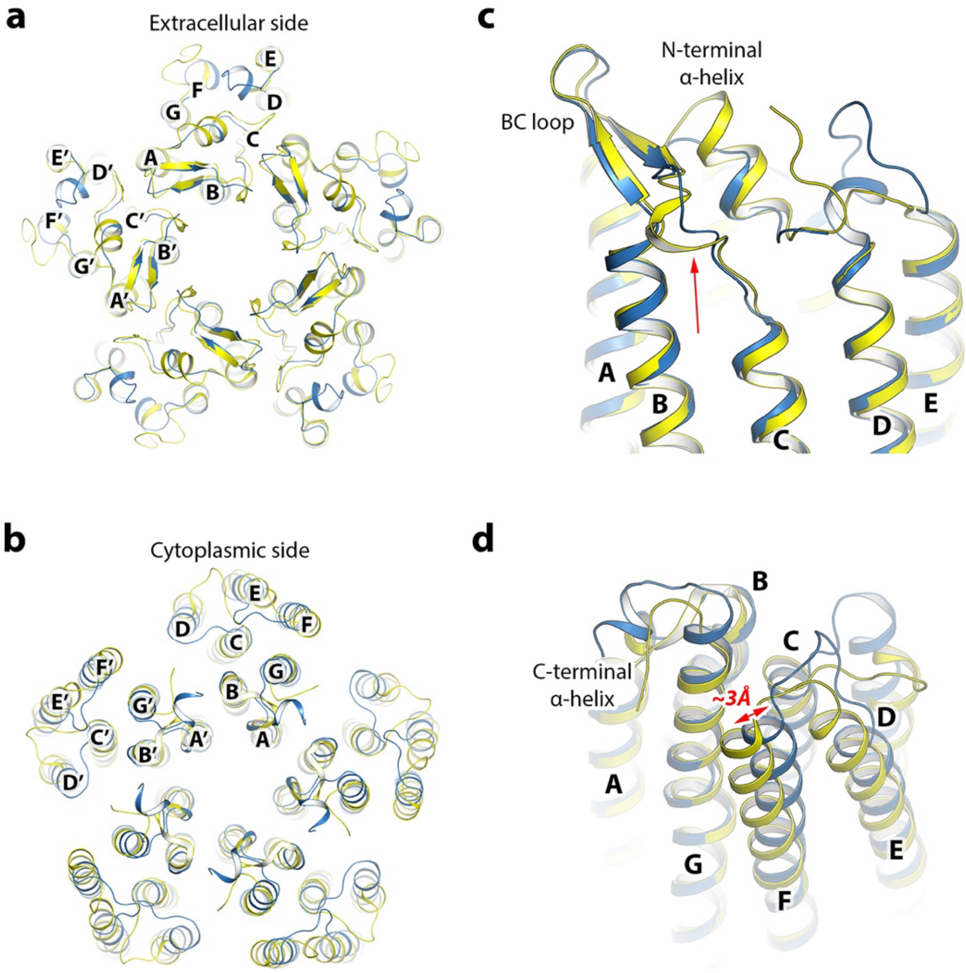

Fig. S11. Comparison between the *Er*NaR and KR2 structures. a. Alignment of the pentamers of *Er*NaR (blue) and KR2 (yellow). View from the extracellular side. **b.** Alignment of the pentamers of *Er*NaR (blue) and KR2 (yellow). View from the cytoplasmic side. **c.** The extracellular side of the *Er*NaR and KR2 protomers. The red arrow indicates the end of the BC loop organized differently in KR2 and *Er*NaR. **d.** The cytoplasmic side of the *Er*NaR and KR2 protomers. The red arrow indicates the difference in the position of the cytoplasmic end of helix F in the *Er*NaR and KR2.

a

No	Charge	Protonations			Calc. Max		GS energy
		D105	D242	E64	[nm]	[eV]	[kcal/mol]
1	-3				459.54	2.70	+731.71
2	-2	x			433.24	2.86	+318.16
3	-2		x		424.15	2.92	+330.74
4	-2			x	442.32	2.80	+333.83
5	-1	x		x	579.78	2.14	+10.67
6	-1		x	x	513.5 (515.0)	2.42 (2.41)	0.00
7	-1	x	x		580.70	2.14	+8.77

b

Total	SAPT0	interaction	-26.00 kcal/mol
Electrostatics [kcal/mol]	Steric repulsion [kcal/mol]	Polarisation [kcal/mol]	London dispersion [kcal/mol]
-35.91	39.75	-22.04	-4.36

c

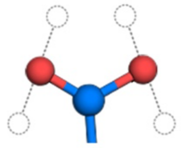

d

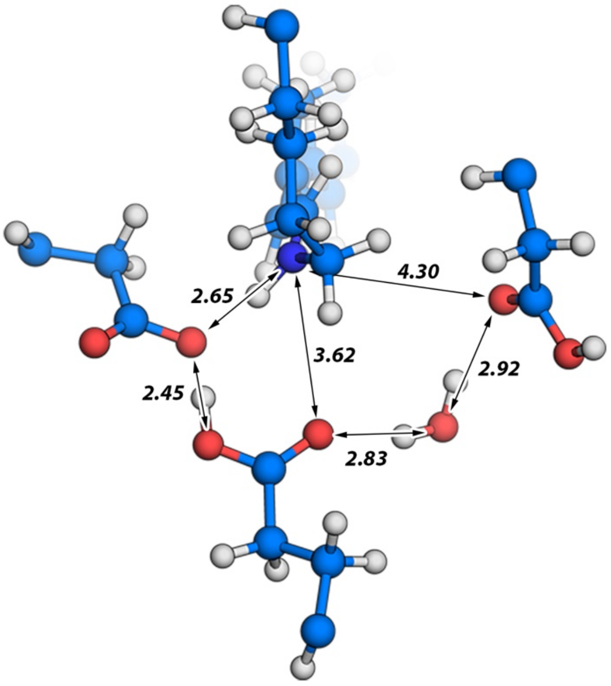

Fig. S12. Overview of the QM/MM simulations. a. Various protonation patterns with the specification of individual protonation of counterions, excitation energies, total charge of carboxylic acids, and Ground State (GS) energies (values of GS energies were normalized with respect to the value of the lowest energy). Model 6 represents a protonation pattern the closest in agreement with the experiment (~530 nm, i.e., 2.34 eV), as well as the lowest ground state energy compared to energies of "-1" protonation systems. The wavelength in brackets corresponds to the inclusion of arginine (R98) for the quantum mechanics (QM) region, which slightly improves the agreement with the recorded spectrum. **b.** SAPT0 interaction energy components of D105 and E64 sidechains of model 6 of *Er*NaR. **c.** Orientations of the hydrogen atom for each of the three counter ions considered in QM/MM simulations. **d.** The visualization of model 6 and relative distances.

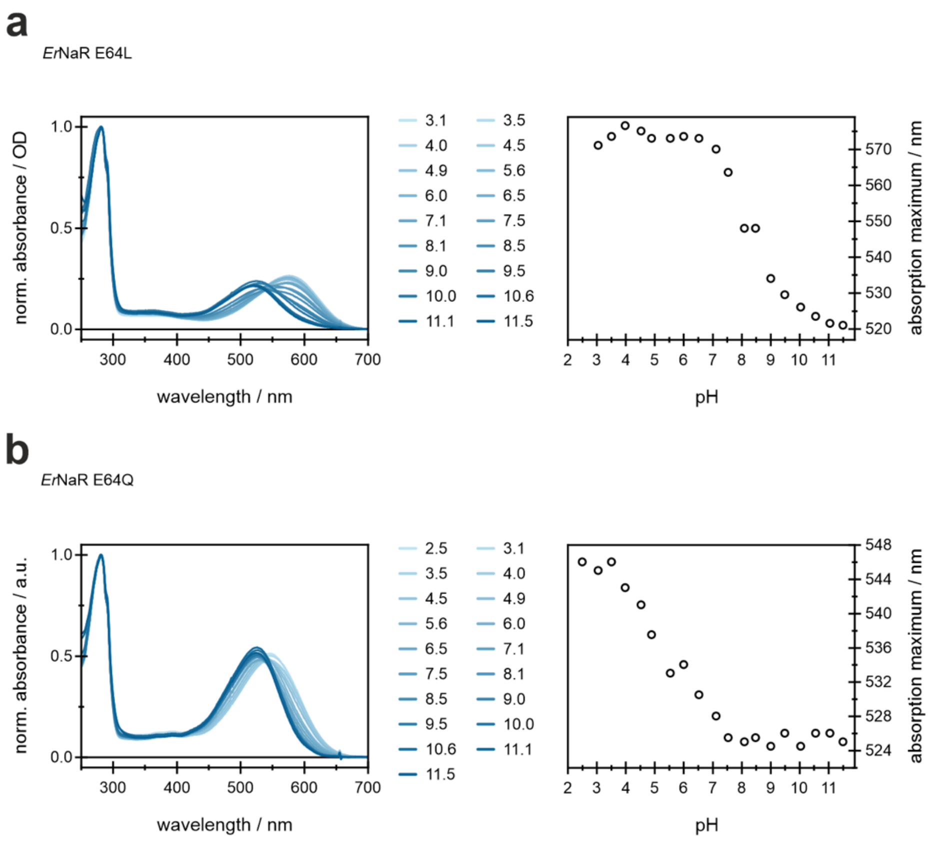

Fig. S13. pH titration of detergent-solubilized *Er*NaR mutants E64L and E64Q. Normalized absorption spectra of **a.** *Er*NaR E64L and **b.** *Er*NaR E64Q. The absorption spectra have been normalized to the absorbance at 280 nm. Furthermore, the pH dependent position of the absorption maximum is shown to illustrate the pH dependent shift of the absorption maximum of both mutants.

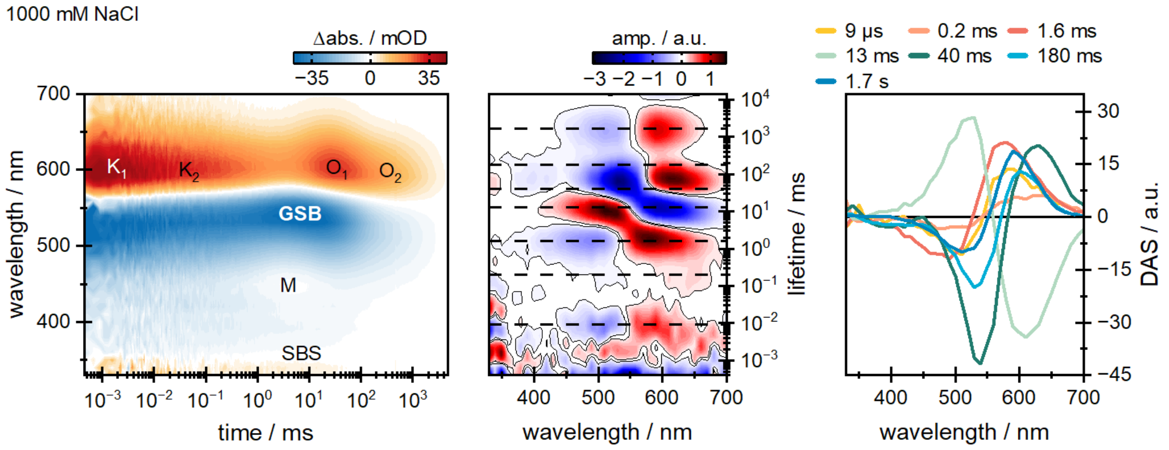

Fig. S14. Comparison of lifetime distribution analysis and global lifetime analysis. Exemplary comparison of the results of lifetime distribution analysis (LDA) and global lifetime analysis (GLA) to show the complementarity of both analyzation methods for time-resolved spectroscopic datasets. The via GLA obtained lifetimes have been highlighted as dashed lines in the LDM to show the comparability of both analyzation methods.

Supplementary Tables

Table S1. List of oligonucleotides for mRNA production.

**Name of mRNA**	**Forward primer**	**Reverse primer**
C2C1-*Er*NaR-TS-FLAG	taatacgactcactatagggccaccatggactatgggggag	tcatcacttgtcatcatcgtccttgtagtccacgttgatgtcgatctggt
EYFP	taatacgactcactatagggccaccatggtgagcaagggcgaggagctg	tcatcacttgtacagctcgtccatgccgagagtgatcc

Table S2. Composition of extracellular and intracellular solutions and liquid junction potentials in whole cell patch-clamp experiments. All concentrations are given in mM. LJPs are in mV. The pH values of all solutions were very close to those of the buffers used but were adjusted if needed with 1M HCl or 1M KOH. Abbreviations: HEPES, 4-(2-hydroxyethyl)-1-piperazineethanesulfonic acid; EGTA, Ethyleneglycol- bis(β-aminoethyl)-N,N,N′,N′-tetraacetic Acid; Tris, tris(hydroxymethyl)aminomethane; LJP, liquid junction potential.

	**Extracellular solution**	**Intracellular solutions**								
		**130 mM Na^+ pH 7.5**	**20 mM Na^+ pH 7.5**	**0 mM Na^+ pH 7.5**	**130 mM Na^+ pH 5.0**	**20 mM Na^+ pH 5.0**	**0 mM Na^+ pH 5.0**	**130 mM Na^+ pH 9.0**	**20 mM Na^+ pH 9.0**	**0 mM Na^+ pH 9.0**
**LJP (extracellular solution)**	-	0.5	-7.3	-7.9	0.6	-5.3	-6.6	-0.4	-7.5	-8
**NaCl**	110	110	-	-	110	-	-	110	-	-
**Arginine-Cl**	-	-	110	110	-	110	110	-	110	110
**$MgCl_2$**	2	2	2	2	2	2	2	2	2	2
**HEPES [NaOH] pH 7.5**	10	-	-	-	-	-	-	-	-	-
**HEPES [KOH] pH 7.5**	-	10	10	10	-	-	-	-	-	-
**EGTA [NaOH] pH 7.2**	-	10 20 mM of Na^+	10 20 mM of Na^+	-	10 20 mM of Na^+	10 20 mM of Na^+	-	10 20 mM of Na^+	10 20 mM of Na^+	-
**EGTA [Tris] pH 7.2**	-	-	-	10	-	-	10	-	-	10
**K-citrate buffer pH 5.0**	-	-	-	-	10	10	10	-	-	-
**Tris buffer pH 9.0**	-	-	-	-	-	-	-	10	10	10

Table S3. Cryo-EM data collection, refinement, and validation.

	**4.3**	**8.0**
**PDB ID**	8QR0	8QQZ
**Data collection and processing**		
Voltage (kV)	300	300
Electron exposure ($e^-/Å^2$)	50.0	60.0
Defocus range (μm)	−0.9 to −2.7	−0.5 to −2.0
Pixel size (Å)	0.836	0.418
Micrographs collected	5,688	7,318
Symmetry imposed	C5	C5
Initial dataset (# of particles)	6,363,353	5,753,452
Final dataset (# of particles)	555,294	437,017
Map resolution (Å) $FSC_{01.43}$	2.50	2.63
**Refinement**		
Initial model used	Alphafold	Alphafold
Map-sharpening *B* factor ($Å^2$)	111.4	111.0
No. atoms		
Protein	10,735	10,801
Retinal	100	100
Lipid	618	775
Water	145	195
B-factors ($Å^2$)		
Protein	34.1	24.8
Retinal	23.6	13.5
Lipid	54.9	59.6
Water	41.0	30.6
R.m.s. deviations		
Bond lengths (Å)	0.013	0.012
Bond angles (°)	1.787	1.633
**Validation**		
MolProbity score	1.78	1.54
Clash score	5.64	4.98
Poor rotamers (%)	4.26	2.34
Ramachandran plot (%)		
Favored	98.88	99.25
Allowed	1.12	0.75
Outliers	0.00	0.00
Model to map fit CC	0.92	0.90

Table S4. X-ray crystallography data collection and structure refinement statistics on *Er*NaR.

	**pH 4.6**	**pH 8.8**
**PDB ID**	8QLE	8QLF
**Data collection**		
Space group	P 61 2 2	P 61 2 2
Cell dimensions		
a, *b*, *c* (Å)	53.40, 53.40, 365.05	53.38, 53.38, 364.84
a, b, g (°)	90, 90, 120	90, 90, 120
Resolution (Å)	60.842-1.664 (1.763-1.664)*	46.230-1.707 (1.875-1.707)*
R_{pim}, %	5.2 (116.1)	3.8 (51.8)
I / s*I*	11.0 (0.7)	13.8 (1.6)
Completeness (%)	94.3 (78.4)	94.7 (69.0)
Redundancy	35.9 (30.2)	37.6 (34.9)
**Refinement**		
Resolution (Å)	20-1.70	20-1.71
No. reflections	28,309	25,066
R_{work} / R_{free}	21.2/24.8	18.3/21.8
No. atoms		
Protein	2186	2184
Retinal	20	20
Lipid	147	214
Water	87	112
B-factors (Å^2)		
Protein	29.9	29.2
Retinal	22.4	21.2
Lipid	49.8	55.5
Water	39.3	40.5
R.m.s. deviations		
Bond lengths (Å)	0.002	0.003
Bond angles (°)	1.025	1.046

*Values in parentheses are for the highest-resolution shell.
