# Supplementary material for: A subgroup of light-driven sodium pumps with an additional Schiff base counterion
Source: Nat Commun. 2024 Apr 10;15:3119. doi: 10.1038/s41467-024-47469-0 (PMC11006869; doi:10.1038/s41467-024-47469-0)
Supplement: Supplementary file 3 — Description of Additional Supplementary Files [file 41467_2024_47469_MOESM3_ESM.pdf]

Description of Additional Supplementary Files

File Name: Supplementary Movie 1
Description: Cryo-EM maps for the pentameric model of ErNaR at pH 8.0.

File Name: Supplementary Movie 2
Description: Cryo-EM maps for the pentameric model of ErNaR at pH 4.3.
